# National Family Caregiver Support Program Participants' Recommendations to Boost Caregiver Supports

**DOI:** 10.1093/geroni/igab046.3416

**Published:** 2021-12-17

**Authors:** Heather Menne, Natalie Mulmule, Angela Gasdaska, Emily Costilow, Kristen Robinson

**Affiliations:** 1 RTI International, Washington, District of Columbia, United States; 2 RTI International, RTI International, North Carolina, United States; 3 DLH/SSS, Silver Spring, Maryland, United States

## Abstract

For more than 20 years, family caregivers have been supported through the National Family Caregiver Support Program (NFCSP) of the Older Americans Act (Title IIIE). The NFCSP provides information to caregivers about available services; assistance in gaining access to services; counseling, support groups and caregiver training; respite care; and supplemental services. In the 2019 National Survey of Older Americans Act Participants, 1,909 NFCSP caregivers were asked “What recommendations do you have for improving the service?” The resulting 748 open-ended responses were thematically coded. The thematic analysis yielded six major themes: Additional Resources, Staffing, Communication, Care Coordination, Quality of Services, and Eligibility. Sub-themes were identified for Additional Resources and Staffing. The most common sub-themes for Additional Resources were requests for more help or services (e.g., grocery shopping), increased funding or financial assistance, and more service hours (e.g., overnight or holiday care). The most common sub-theme for Staffing was the need for consistent staffing due to high turnover of staff. Chi-Squared tests and Fisher’s Exact tests indicated that there were no significant associations between any of the recommendation themes and the gender of the caregiver, employment status of the caregiver, or whether the care recipient has Alzheimer’s or dementia. Many of the themes align with results from a recent RAISE Family Caregiving Advisory Council Report. Recommendations from both sets of findings indicate ways that programs, services, and policies can be enhanced to support the needs of care recipients and caregivers.

